# *Voces de la frontera*/Voices from the Border: Using Case Studies of Pregnancy, Birth and Parenting along the U.S.–Mexico Border to Identify Shared Measures of Success

**DOI:** 10.1007/s10995-017-2375-z

**Published:** 2017-12-02

**Authors:** Katherine Selchau, Maricela Babuca, Kara Bower, Yara Castro, Araceli Flores, Jonah O. Garcia, Maria Lourdes F. Reyes, Yvonne Rojas, Laura Shattuck

**Affiliations:** 1 PCI U.S. & Border Programs, California Border Healthy Start+, Project Concern International (PCI), 4305 University Ave, Ste., 345, San Diego, CA 92105 USA; 2 Santa Cruz County Healthy Start, Mariposa Community Health Center, 1852 N. Mastick Way, Nogales, AZ 85621 USA; 3 Welcome Baby Program, Ben Archer Health Center, 1600 Thorpe Rd, Las Cruces, NM 88012 USA; 4 Healthy Start Laredo, BCFS Health and Human Services, 7019 Village Blvd., Suite 205, Laredo, TX 78041 USA; 5 Healthy Start Program, La Clinica de Familia, 575 South Alameda Blvd., Las Cruces, NM 88005 USA; 6 California Border Healthy Start+, Project Concern International (PCI), 4305 University Ave, Ste. 345, San Diego, CA 92105 USA

**Keywords:** Pregnancy, Health care access, Medical home, Social determinants of health, U.S.–Mexico border

## Abstract

*Purpose* This research analyzes the cases of five women living along the U.S.–Mexico border who overcame challenges during pregnancy or parenting with the support of a federally funded Healthy Start program, designed to eliminate disparities in perinatal health in disadvantaged communities with the poorest birth outcomes. Study objectives were to: (1) identify common factors that affect healthy maternal and child outcomes for Healthy Start participants; and (2) identify a shared definition of what success looks like for Healthy Start participants and opportunities for further study. *Description* Five border Healthy Start sites (CA, AZ, NM, and TX) contributed case stories from participants who had overcome access barriers to achieve positive pregnancy, birth or parenting outcomes. Case studies were collected using review of successful participant cases and non-structured interviews by Healthy Start staff, and analyzed using participatory methods and thematic analysis. *Assessment* Common barriers were: lack of insurance; isolation or unsupportive family relationships; timidness and lack of self-advocacy. Healthy Start programs have been successful in securing supportive relationships through the community health worker model; reducing isolation; obtaining insurance access and a medical home; building self-advocacy skills; and supporting participants to pursue their goals. *Conclusion* Identified barriers are in line with available literature on health care access and provide a U.S.–Mexico border-specific view. The Healthy Start model is effective at helping women to overcome barriers. Learning from this research may contribute to development of shared measures for more impactful evaluation of Healthy Start and similar programs.

## Significance

Access to health care is a barrier for women along the U.S.–Mexico border, contributing to high rates of unintended pregnancies (Kost 2010), late prenatal care (McDonald et al. 2013), and other poor reproductive health outcomes. Compounding factors include poverty and isolation among largely immigrant populations. Healthy Start programs play a role in overcoming fear and isolation, supporting access to healthcare and legal supports, and improving women’s ability to self-advocate and pursue their goals. This study contributes examples of how addressing these factors may improve pregnancy and birth outcomes for families.

## Purpose

The Healthy Start Initiative is a program of the U.S. Department of Health and Human Services to eliminate disparities in perinatal health in communities with the poorest birth outcomes. Healthy Start projects nationwide provide comprehensive perinatal case management for vulnerable pregnant and interconceptional families to improve access to quality services, key prevention and health promotion messages, and have common performance measures related to maternal and child health. U.S.–Mexico border-based Healthy Starts utilize the community health worker (CHW) model for home visiting and have adopted similar curricula to empower women with life skills and mental health resilience.

**Fig. 1:**
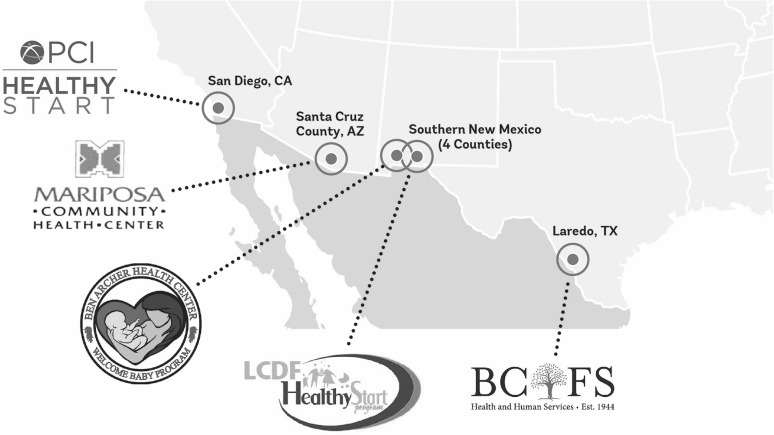
Map of Healthy Start Border Alliance sites

The Healthy Start Border Alliance (HSBA) brings together five U.S.–Mexico border-based Healthy Start projects, in San Diego, CA; Santa Cruz County, AZ; four counties in Southern NM (two projects); and Webb County, TX (Fig. 1). Formed in 2014, the group is aligning curricula and measures to better assess and accelerate its impact on maternal and child health among underserved border communities, a process which this study aims to inform.

Populations living along the U.S.–Mexico border experience myriad challenges that contribute to diminished health and well-being. If the border became the 51st U.S. State, it would rank last in the percentage of people covered by health insurance, last in per capita income, and first in numbers of children who are living in poverty and are uninsured (National Rural Health Association 2010).

The female population in the border region is younger than the overall U.S. population (23% under 15 years in 2007 vs. 19.4% of the U.S.), and are more than twice as likely to live under the federal poverty level (18.8 vs. 8.8%; U.S. Department of Health and Human Services [DHHS] 2009). They have higher fertility rates, higher rates of adolescent and teen pregnancy and late prenatal care (McDonald et al. 2013); as well as higher rates of unintended pregnancies (Kost 2010).

This multiple case study analyzes factors contributing to successful outcomes among five women living along the U.S.–Mexico border who overcame challenges during pregnancy, childbirth and parenting, with the support of Healthy Start. The study’s objectives were to (1) identify common factors that affect healthy maternal and child outcomes for Healthy Start participants; and (2) identify a shared definition of what success looks like for Healthy Start participants and opportunities for further study. This study is not intended to evaluate Healthy Start’s impact on target population outcomes, but to improve understanding of contributors to success and thereby improve future project interventions and evaluations.

## Description

Program leads from each HSBA site, in consultation with staff, selected cases which they felt truly captured the type of success that border Healthy Starts are trying to achieve, and which met the following inclusion criteria: subject over 18 years; was enrolled in Healthy Start for at least one of the last 3 years; overcame healthcare access barriers with the support of Healthy Start; and achieved positive pregnancy, birth, or parenting outcomes somehow attributable to Healthy Start.

Each site identified one case that met all inclusion criteria and offered the richest opportunity for analysis. Non-structured interviews were used to accommodate rich exploration of women’s stories, as well as probing for unique factors in the participants’ experiences. Interviews were conducted in the subject’s preferred language by bilingual staff, and all provided informed consent to use their information prior to inclusion in the study. Each project site used client charts to confirm participant stories.

Analysis was modelled after *Most Significant Change*, a participatory evaluation methodology used to identify unexpected outcomes not already defined by existing evaluation protocols, and shared values that prevail across groups (Davies and Dart 2005). While each site easily identified cases they considered successful based on Healthy Start performance indicators, this methodology helped to identify what specifically was the contributor to their success. Cases were analyzed by the program leads from each site, first individually to explore the questions “What changed?” and “What was Healthy Start’s role?”, and then as a group to explore “What commonalities exist in the way we each define success?” and “What is Healthy Start’s role across programs in achieving that success?”

Identification and analysis of common themes drew upon broad content analysis, exploration and discussion rather than content coding and frequency reporting. Themes, defined as topics that arose in at least two case stories, were identified and discussed by the group. Factors identified as key contributors to success were those themes that emerged most prominently across the case stories and which the group agreed best illustrated Healthy Start’s role in achieving success with participants.

## Results

**Fig. 2:**
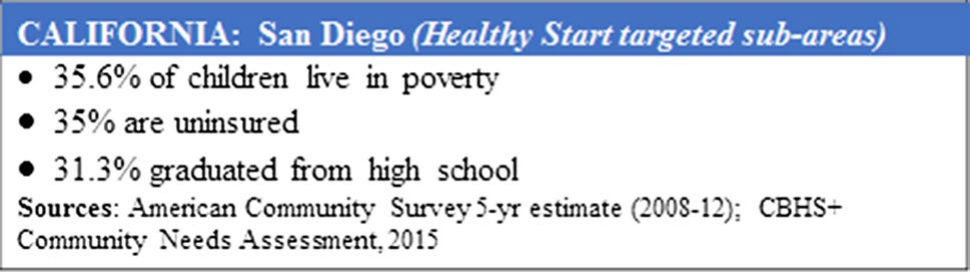
*Mariana, 24*, fled to California: San Diego county statistics. Source: American Community Survey 5-yr estimate (2008-12); CBHS+ Community Needs Assessment, 2015

**Fig. 3:**
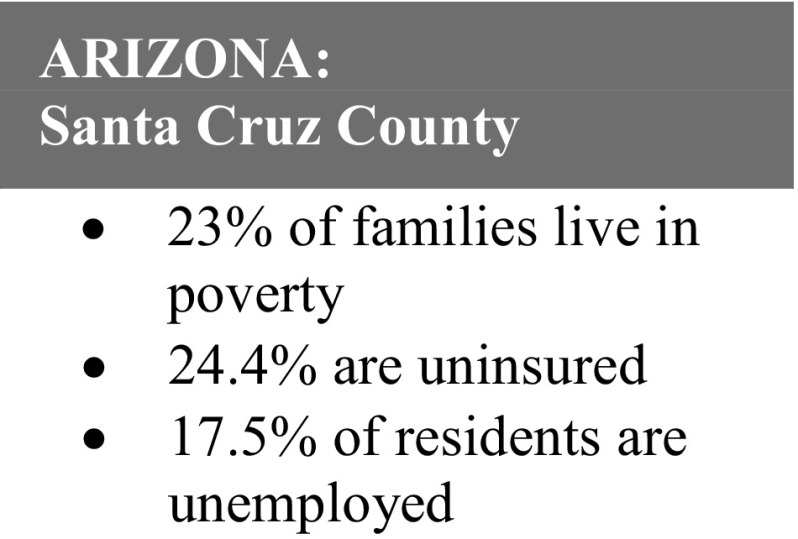
Arizona: Santa Cruz county statistics. Source: U.S. Census Bureau, 2010; Nogales Primary Care Statistical Profile

**Fig. 4:**
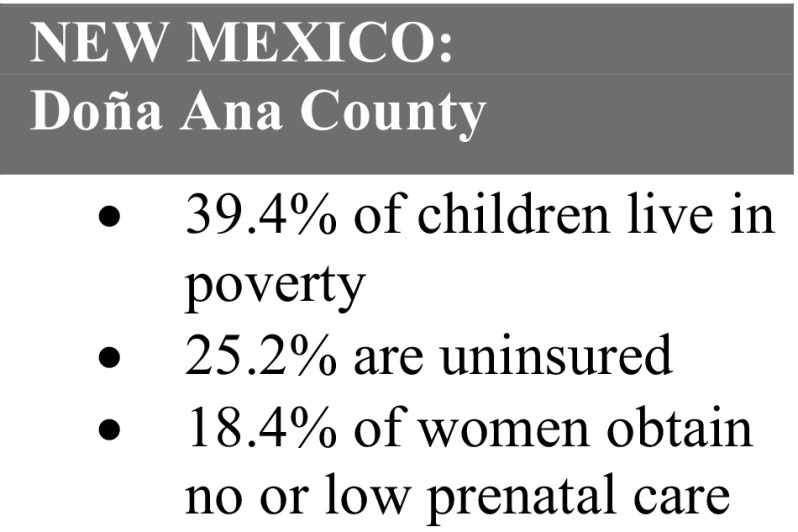
New Mexico: Doña Ana county statistics. Source: New Mexico Department of Health Primary Care Statistical Profile

**Fig. 5:**
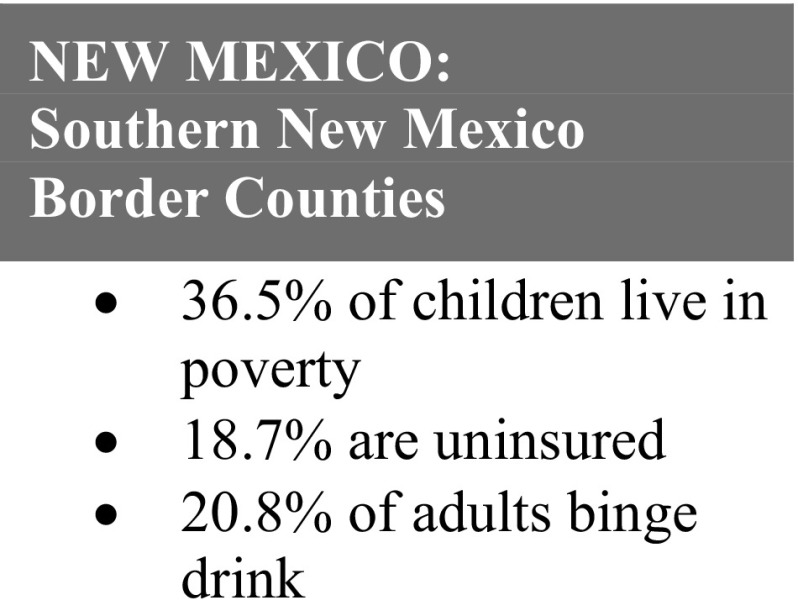
New Mexico: Southern New Mexico statistics. Source: New Mexico Department of Health (2007-2009)

**Fig. 6:**
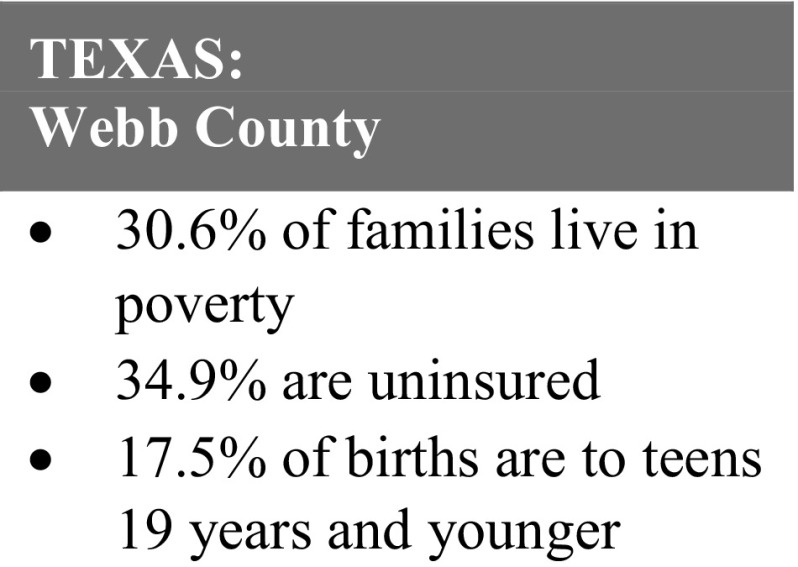
Texas: Webb county statistics. Source: Texas Department of State Health Services, 2013

Top themes identified were improving access to insurance and medical home, reducing isolation, developing self-advocacy skills, and pursuing educational/career goals. Below are brief summaries of the case stories. All names have been changed to pseudonyms. Below are brief summaries of the case stories, each preceded by relevant area statistics (Figs. 2, 3, 4, 5, 6). All names have been changed to pseudonyms.

*Mariana, 24*, fled to California from another state after escaping an abusive relationship. She spoke no English, and collected cans and bottles to make ends meet. She was well into her pregnancy when she went to a community clinic and was referred to Healthy Start.

Healthy Start provided her with case management, prenatal education, and supported her enrollment in English classes. When her ex-boyfriend showed up at her birth and convinced her to give him another chance, the abuse soon resumed. Mariana’s Healthy Start home visitor was the only person she trusted, and worked alongside her until she got the courage to report him to the police and safely escape from the relationship. Healthy Start provided her with emergency assistance for her and her baby so that she could continue to make her medical appointments, attend school and a domestic violence support group.

Since then, Mariana has continued going to school, has attended Healthy Start’s classes in depression prevention, life skills and community health worker training, and has worked as a nanny. In January, 2016, she completed her licensure as certified nurse assistant. She is in the process of obtaining a U-Visa for domestic violence victims to stay and work in the U.S.

“I am so thankful to the Healthy Start program. I always felt supported and guided by my home visitor and I knew I was not alone, even when I was so afraid. It has not been easy as a single mother, but I know that my daughter and I have a chance for a better future.”

*Rosa, 38*, joined Healthy Start during the last trimester of her fifth pregnancy. Though she was a legal U.S. resident, she did not qualify for health insurance because she had been in the U.S. for < 5 years. She and her children lived in poverty and had no healthcare coverage; as a result she sought prenatal care in Mexico.

Rosa’s friend referred her to Healthy Start, and her home visitor connected her to affordable prenatal care through a community health center, helped to her apply for health insurance for her children, provided health education, and taught her how to navigate other needed services.

Rosa’s child is now 18 months and both continue to be a part of the Healthy Start family. Rosa has become an advocate for Healthy Start, referring numerous women to the program.

“Healthy Start helped me to find my voice. Because of the Healthy Start program I know how to speak up if I feel that my or my children’s wellbeing could be harmed.”

*Cristina, 26*, was brought to the U.S. as a young child when her parents came in search of a better life. Her parents worked hard to obtain permanent residency, and her only responsibility was to finish high school, go to college, and acquire the knowledge and skills needed to gain meaningful employment. When she became pregnant at 19, she felt overwhelmed by the shame and disappointment it would bring to her family.

Cristina did not have health insurance and was nearing her third trimester when she enrolled in Healthy Start. She was too afraid to tell her parents about the pregnancy. With Healthy Start’s support, she started seeing a doctor, obtained health insurance and WIC, and gained the confidence to ask her family for support.

Today, Cristina is the mother of two healthy children and is proud to be fulfilling her family’s “American dream.” She had the chance to travel to Washington D.C. to tell her story to Senators and Congressman during the National Healthy Start Conference. She has since been employed by the Healthy Start program, where she received training to become a community health worker, and is pursuing a degree in early childhood. She and her husband are in the process to obtain permanent residency.

“I have been empowered to pursue my dreams and want to empower other women to follow theirs as well. I am forever grateful for the care and support received from the Healthy Start Program.”

*Isabel, 36*, is a recovering alcoholic and mother of a healthy 2-year-old daughter. When she joined Healthy Start, she did not have a job, was renting a small apartment, and screened high for post-partum depression. Her HS home visitor connected her to behavioral health services, educated her in her role as a mother to support the development of her child, and helped her to establish family goals.

With Healthy Start’s continuous support and encouragement, Isabel has fulfilled her goals to remain sober, to get a job to provide for her family and enroll her daughter at a childcare center, and is in the process of buying a house for her and her daughter. Isabel no longer feels depressed and actively sees a behavioral health therapist.

“I like having someone to talk to that doesn’t judge me. [Healthy Start] has helped me to move past my problems, I am now able to tell people my story without being ashamed.”

*Ana, 18*, emigrated from Mexico as a small child. She shares a small mobile home with her extended family in a *colonia* near the Texas–Mexico border, which lacks basic services such as potable water and sewer systems, electricity, or paved roads. Her family visits a water pump several times a week to bring back clean water for bathing, laundry, and cooking.

Ana became pregnant while still in high school, and had no healthcare coverage and no access to healthcare. When she joined in 2013, Healthy Start assisted with her State benefit application and obtained prenatal care for her at a mobile medical unit. The program provided her transportation to her appointments to reduce school absences and help her keep focused on her goal of graduating high school.

As a result of her participation in Healthy Start, Ana obtained prenatal care, health insurance, and earned her high school diploma soon after giving birth to a healthy baby girl. She has attended classes on nurturing, safe sleep practices, reproductive life planning, and is currently pursuing a degree in accounting at the local community college.

“The Healthy Start program is helpful in many ways. I have become the mother I always knew I could be. I am always learning new things and now I set goals for myself to keep me focused.”

## Discussion

These case stories show how Healthy Start programs extend support beyond participants’ healthcare needs to encompass social, economic and other issues they face. Healthy Start benchmarks allow programs to measure specific perinatal outcomes, adoption of improved behaviors or access to specific services. However, programs might consider adopting evaluation tools that can better measure Healthy Start’s success in addressing the social determinants of health, including educational attainment and employment, demonstrated to be associated with improved birth outcomes (Luo et al. 2006; Olson et al. 2010; Scharber 2014). PCI’s *Lives Changed Index* incorporates validated instruments as well as subscales developed by PCI and measures material circumstances, financial security, and access to basic needs (PCI 2013). The tool has been piloted in the border setting and may offer a powerful opportunity to measure the impact of Healthy Start interventions on material and socioeconomic circumstances.

Isolation and lack of family support influenced subjects in delaying care, consistent with pregnancy care literature (Higgins et al. 1994; Webster et al. 2000). Trust is an important indicator of quality in patient–provider relationships and predicts adherence to protective health behaviors (Sheppard et al. 2004). While Healthy Start’s direct role in increasing relationship support varied among programs, case studies provided examples of how access to a trusted, supportive partnership with a Healthy Start home visitor was operative in reducing fear and isolation for participants. This provided the backdrop for subjects to overcome personal challenges and build supportive relationships in their lives. Healthy Start programs screen participants for domestic violence, and two HSBA sites use the *Relationship Assessment Tool* (WEB), an evidence-based screening tool that measures healthy and safe relationships and is designed to help clients and patients recognize how unhealthy relationships and abuse can negatively impact their health outcomes (Smith et al. 1994). Alignment of relationship screening tools across projects could allow Healthy Start programs to better study their success in promoting supportive relationships that can improve health outcomes.

An important theme was empowering participant self-advocacy to seek care and pursue individual goals. Self-advocacy is a protective factor for health (Wallerstein 2002) and improved birth outcomes (Rini et al. 1999), is measurable through several validated tools (Brashers et al. 1999), and should be explored further as a potential cornerstone objective of Healthy Start projects. Subjects who, as a result of their experience in the program, became CHWs, outreach volunteers, and one who even represented Healthy Start in Washington, D.C., offer inspiring examples of how Healthy Start has empowered vulnerable women with the skills and passion to make a difference in their communities. Three HSBA sites are piloting the use of the *Personal Progress Scale* (PPS-R), a validated tool that measures wellbeing, self-esteem, sense of belonging, agency and ability to make decisions in the household/community (Johnson et al. 2005). As HSBA sites collectively scale common curricula in life skills, financial literacy and mental health resilience, the use of these instruments will help them to better capture the impact these interventions have on multiple aspects of participants’ lives.

### Study Limitations

Although sites provide similar service models, absence of a truly standardized protocol across sites contributes to the limited generalizability of study findings. Selected case studies may not be representative of the overall target population. Any associations identified through the case studies could not have been analyzed statistically, therefore any trends identified may be considered for subsequent research to identify relationships through rigorous statistical analysis.

## Conclusions

Common barriers or obstacles to care seeking and healthy birth outcomes included lack of insurance; isolation or unsupportive relationships; timid-ness and lack of self-advocacy. These are in line with available literature on health care access and provide a U.S.–Mexico border-specific view.

The contribution of border Healthy Starts in overcoming these barriers consisted in securing trusting, supportive relationships through the CHW model, reducing isolation, obtaining insurance access and a medical home, building participants’ self-advocacy skills to speak up for themselves and their children, and supporting them to improve their socioeconomic status and pursue goals. These can be better developed into shared measures for evaluation.
